# Supplementary material for: Diffusion MRI anisotropy in the cerebral cortex is determined by unmyelinated tissue features
Source: Nat Commun. 2022 Nov 5;13:6702. doi: 10.1038/s41467-022-34328-z (PMC9637141; doi:10.1038/s41467-022-34328-z)
Supplement: Supplementary file 3 — Reporting Summary [file 41467_2022_34328_MOESM3_ESM.pdf]

nature portfolio

Corresponding author(s): Colin Reveley, David Leopold

Last updated by author(s): 2022/06/21

Reporting Summary

Nature Portfolio wishes to improve the reproducibility of the work that we publish. This form provides structure for consistency and transparency in reporting. For further information on Nature Portfolio policies, see our Editorial Policies and the Editorial Policy Checklist.

Statistics

For all statistical analyses, confirm that the following items are present in the figure legend, table legend, main text, or Methods section.

n/a	Confirmed	
☐	☒	The exact sample size (*n*) for each experimental group/condition, given as a discrete number and unit of measurement
☐	☒	A statement on whether measurements were taken from distinct samples or whether the same sample was measured repeatedly
☐	☒	The statistical test(s) used AND whether they are one- or two-sided<br>*Only common tests should be described solely by name; describe more complex techniques in the Methods section.*
☒	☐	A description of all covariates tested
☒	☐	A description of any assumptions or corrections, such as tests of normality and adjustment for multiple comparisons
☐	☒	A full description of the statistical parameters including central tendency (e.g. means) or other basic estimates (e.g. regression coefficient) AND variation (e.g. standard deviation) or associated estimates of uncertainty (e.g. confidence intervals)
☐	☒	For null hypothesis testing, the test statistic (e.g. *F*, *t*, *r*) with confidence intervals, effect sizes, degrees of freedom and *P* value noted<br>*Give P values as exact values whenever suitable.*
☒	☐	For Bayesian analysis, information on the choice of priors and Markov chain Monte Carlo settings
☒	☐	For hierarchical and complex designs, identification of the appropriate level for tests and full reporting of outcomes
☐	☒	Estimates of effect sizes (e.g. Cohen's *d*, Pearson's *r*), indicating how they were calculated

Our web collection on statistics for biologists contains articles on many of the points above.

Software and code

Policy information about availability of computer code

Data collection	MRI data were collected using commercial scanner and software (Bruker's ParaVision 5.1). Histology data were collected using commercial Ziess Axioscan software.
Data analysis	MRI data was analysed using Tortoise v3, FSL 5.0, imageJ 2.0, and custom matlab scripts, including some third party scripts. Custom scripts are available at 10.6084/m9.figshare.21342341

For manuscripts utilizing custom algorithms or software that are central to the research but not yet described in published literature, software must be made available to editors and reviewers. We strongly encourage code deposition in a community repository (e.g. GitHub). See the Nature Portfolio guidelines for submitting code & software for further information.

Data

Policy information about availability of data

All manuscripts must include a data availability statement. This statement should provide the following information, where applicable:

- Accession codes, unique identifiers, or web links for publicly available datasets
- A description of any restrictions on data availability
- For clinical datasets or third party data, please ensure that the statement adheres to our policy

Raw data is available at https://doi.org/10.6084/m9.figshare.21342473 preprocessed data and analysis code at https://doi.org/10.6084/m9.figshare.21342341 The paxinos atlas is available at https://marmosetbrainmapping.org/atlas.html#v1

Field-specific reporting

Please select the one below that is the best fit for your research. If you are not sure, read the appropriate sections before making your selection.

- [x] Life sciences
- [ ] Behavioural & social sciences
- [ ] Ecological, evolutionary & environmental sciences

For a reference copy of the document with all sections, see nature.com/documents/nr-reporting-summary-flat.pdf

Life sciences study design

All studies must disclose on these points even when the disclosure is negative.

Sample size	By design, all analysis was carried out using a within subject paradigm on a single ex-vivo brain sample. We did not employ statistical methods to determine sample size, since we knew one sample brain is sufficient. There were two main reasons for this: First, the goal of the experiments was to understand the nature of the MRI scans, not the nature of the brain sample. The study assessed whether different forms of measurement give comparable results, rather than assessing the properties of the subject per se. Second, the fact the sample was dead - and therefore a biologically inert object - meant that its properties were not expected to change between measurements due to endogenous noise or biological processes.<br><br>The study design assessed the similarity of different data acquisition modalities from a fixed set of locations in a single dead brain, rather than comparing data from different subjects or samples under different experimental conditions. Our results address the extent to which measurements taken from the same unchanging object, but via different methods, covary.
Data exclusions	Histological sections were excluded due to damage or poor stain properties
Replication	We compared measurements from 19 histological sections to measurements taken via MRI from the same tissue. These results are reported in detail in the text. We also repeated the MRI scan six times (see Methods). Each scan repetition was comparable and we used an average dataset of all six in our experiments.
Randomization	There was no randomization of subjects, because there were no experimental groups. We did perform a spatial randomization of the measurements we were comparing (Figure 5B).
Blinding	There was no blinding, because there were no experimental groups, and there was no subjective judgment to be made by the experimenter.

Reporting for specific materials, systems and methods

We require information from authors about some types of materials, experimental systems and methods used in many studies. Here, indicate whether each material, system or method listed is relevant to your study. If you are not sure if a list item applies to your research, read the appropriate section before selecting a response.

Materials & experimental systems

n/a	Involved in the study	
[ ]	[x]	Antibodies
[x]	[ ]	Eukaryotic cell lines
[x]	[ ]	Palaeontology and archaeology
[ ]	[x]	Animals and other organisms
[x]	[ ]	Human research participants
[x]	[ ]	Clinical data
[x]	[ ]	Dual use research of concern

Methods

n/a	Involved in the study	
[x]	[ ]	ChIP-seq
[x]	[ ]	Flow cytometry
[ ]	[x]	MRI-based neuroimaging

Antibodies

Antibodies used	rabbit anti MAP-2 primary antibody (188 003, Synaptic Systems, Germany), goat anti-rabbit secondary antibody conjugated to DyLight 680 fluorophore (35569, Invitrogen, Waltham, Ma)
Validation	https://www.sysy.com/product/188003

Animals and other organisms

Policy information about studies involving animals; ARRIVE guidelines recommended for reporting animal research

Laboratory animals	common marmoset, female 5 years old, female 2 years old
Wild animals	no wild animals were used in this study

Field-collected samples	N/A
Ethics oversight	N/A. Ex-vivo specimen case P was from a previously published study.

Note that full information on the approval of the study protocol must also be provided in the manuscript.

Magnetic resonance imaging

Experimental design

Design type	N/A
Design specifications	N/A
Behavioral performance measures	N/A

Acquisition

Imaging type(s)	ex-vivo diffusion MRI
Field strength	7T
Sequence & imaging parameters	Spin-echo diffusion-weighted EPI sequence was used to collect data from a brain sample. Acquisition parameters were described in detail in the method section.
Area of acquisition	left and right hemispheres separately acquired

Diffusion MRI ☒ Used ☐ Not used

Parameters: Number of direction was 60 for the left hemisphere and 126 for the right hemisphere. The b-value was 4800 s/mm−2. No multi-shell, no cardiac gating.

Preprocessing

Preprocessing software	TORTOSE v3
Normalization	no normalization
Normalization template	N/A
Noise and artifact removal	Denoise function included in TORTOISE v3 was used to suppress thermal noise in the preprocessing step.
Volume censoring	N/A

Statistical modeling & inference

Model type and settings	N/A
Effect(s) tested	N/A

Specify type of analysis: ☐ Whole brain ☐ ROI-based ☒ Both

Anatomical location(s): we matched MRI locations to histology taken from the brain sample

Statistic type for inference (See Eklund et al. 2016)	N/A
Correction	N/A

Models & analysis

n/a	Involved in the study
☒	☐ Functional and/or effective connectivity
☒	☐ Graph analysis
☒	☐ Multivariate modeling or predictive analysis
